# 

**Published:** 1923-01

**Authors:** 


					﻿THE
PIONEER DENTAL JOURNAL OF THE UNITED STATES
SEVENTY-SEVENTH YEAR
THE
DENTAL
REGISTER
THE MAGAZINE Of DENTISTRY
Vol. LXXVII JANUARY, 1923 No. 1
A Monthly Journal Devoted to the
Interests of the Dental Profession.
PUBLISHERS
OHIO DENTAL AND SURGICAL DEPOT
CONRAD BLDG., 18 & 20 W. Seventh St, CINCINNATI, O.
Price, $3.00 per year in U. 8. and Canada
in Foreign Countries, $4.00
Price per copy, 80 cetite
ESTABLISHED OCTOBER, 1847
				

